# Parenteral Adjuvant Effects of an Enterotoxigenic *Escherichia coli* Natural Heat-Labile Toxin Variant

**DOI:** 10.3389/fimmu.2013.00487

**Published:** 2014-01-07

**Authors:** Catarina J. M. Braga, Juliana F. Rodrigues, Yordanka Medina-Armenteros, Luís E. Farinha-Arcieri, Armando M. Ventura, Silvia B. Boscardin, Maria E. Sbrogio-Almeida, Luís C. S. Ferreira

**Affiliations:** ^1^Department of Microbiology, Institute of Biomedical Sciences, University of São Paulo, São Paulo, Brazil; ^2^Department of Parasitology, Institute of Biomedical Sciences, University of São Paulo, São Paulo, Brazil; ^3^Division of Technological Development, Butantan Institute, São Paulo, Brazil

**Keywords:** vaccines, adjuvants, heat-labile toxin, HIV-1, p24, intradermal immunization, cytotoxic T cell response

## Abstract

Native type I heat-labile toxins (LTs) produced by enterotoxigenic *Escherichia coli* (ETEC) strains exert strong adjuvant effects on both antibody and T cell responses to soluble and particulate antigens following co-administration via mucosal routes. However, inherent enterotoxicity and neurotoxicity (following intra-nasal delivery) had reduced the interest in the use of these toxins as mucosal adjuvants. LTs can also behave as powerful and safe adjuvants following delivery via parenteral routes, particularly for activation of cytotoxic lymphocytes. In the present study, we evaluated the adjuvant effects of a new natural LT polymorphic form (LT2), after delivery via intradermal (i.d.) and subcutaneous (s.c.) routes, with regard to both antibody and T cell responses. A recombinant HIV-1 p24 protein was employed as a model antigen for determination of antigen-specific immune responses while the reference LT (LT1), produced by the ETEC H10407 strain, and a non-toxigenic LT form (LTK63) were employed as previously characterized LT types. LT-treated mice submitted to a four dose-base immunization regimen elicited similar p24-specific serum IgG responses and CD4^+^ T cell activation. Nonetheless, mice immunized with LT1 or LT2 induced higher numbers of antigen-specific CD8^+^ T cells and *in vivo* cytotoxic responses compared to mice immunized with the non-toxic LT derivative. These effects were correlated with stronger activation of local dendritic cell populations. In addition, mice immunized with LT1 and LT2, but not with LTK63, via s.c. or i.d. routes developed local inflammatory reactions. Altogether, the present results confirmed that the two most prevalent natural polymorphic LT variants (LT1 or LT2) display similar and strong adjuvant effects for subunit vaccines administered via i.d. or s.c. routes.

## Introduction

Type I heat-labile toxins (LTs) produced by some enterotoxigenic *Escherichia coli* (ETEC) strains belong to a family of structurally and immunologically related enterotoxins associated with traveler’s diarrhea (1). LTs consist of one A subunit (LTA) non-covalently bound to the pentameric B subunit (LTB), which is formed by the union of five identical polypeptides. The A subunit has ADP ribosyltransferase activity, and the B subunit targets the protein to glycosphingolipid receptors on the surface of eukaryotic cells (e.g., GM1 ganglioside). After receptor binding, the toxin is internalized and cleaved proteolytically, and the active A1 domain is released into the cytoplasm, resulting in the permanent activation of the Gsα component of adenylate cyclase. The enhanced 3′,5′-cyclic monophosphate (cAMP) levels promote massive ion and water losses from the enterocytes to the intestinal lumen, leading to diarrhea (1).

In addition to their pivotal role in the etiology of ETEC-associated secretory diarrhea, LTs have attracted considerable interest due to their strong **adjuvant** effects observed after co-administration of the toxin with soluble or particulate antigens via mucosal (2–9) or transcutaneous routes (9–11). To increase the safety of LTs as mucosal adjuvants, mutant forms have been generated, including LTK63, which is devoid of ADP-ribosylation activity but partially preserves the adjuvant effects (3, 12–15). However, clinical trial results were disappointing due either to the induction of unacceptable side effects (transient facial paralysis) after intra-nasal administration of LTK63 or to reduced adjuvant effects in subjects immunized with LT-adjuvanted adhesive vaccine patches (10, 11, 15).

Recently, a significant degree of genetic diversity has been detected in the LTs produced by ETEC strains isolated from symptomatic and asymptomatic subjects in Brazil. Sixteen LT types were identified, including LT1, expressed by the reference ETEC H10407 strain and several other ETEC strains of different serotype groups (16). Another LT type, named LT2, represents the second most prevalent natural LT form found among LT-producing ETEC strains. DNA sequencing revealed that LT2 has six polymorphic sites compared to the LT1: five in the A subunit (S190L, G196D, K213E, S224T, and N238D) and one in the B subunit (T75A) (16). LT2 showed both ADP-ribosylation activity and binding to host cell receptors but showed different immunological features compared with the reference LT, particularly with regard to the humoral adjuvant effects by transcutaneous administration (9). The same LT natural variant has also been detected in an ETEC strain isolated from a diarrheic tourist in Japan, which suggest that this LT-encoding gene may have a widespread occurrence (17).

In the present study, we further investigated the immunological features of LT2 in comparison with other known LT forms, including LT1 and LTK63, particularly with regard to the adjuvant effects for both humoral (antibody) and cellular (T cell) responses elicited in mice immunized via parenteral routes (intradermal and subcutaneous) with co-administered recombinant HIV-1 p24 protein as a model antigen.

## Materials and Methods

### Cloning, expression, and purification of the recombinant HIV-1 p24 protein

The coding sequence for the HIV-1 p24 antigen was amplified from the pHXB2 plasmid (GenBank Accession Number K03455) (18) using the following primers: sense 5′-CGAT*G^∧^CTAGC*ATGCAGAACATCCAGGGGCA-3′and antisense 5′-GT*G^∧^AATTC*TCAAGCCAAAACTCTTGCCTTA-3′. The amplified fragment was inserted in frame into the pET28b expression vector (Novagen) between the *Nhe*I and *Eco*RI restriction sites (underlined). BL21(DE3) strain *E. coli* (Invitrogen) were transformed with the expression vector, and after it was carried out screening for positive clones by restriction analysis and DNA sequencing. Induction of the recombinant protein was achieved by addition of isopropyl-β-d-thiogalactopyranoside (IPTG) to the culture for 4 h at 37°C under aeration. The recombinant p24 with an N-terminal histidine tag was purified using a HisTrap™ HP column (GE Healthcare Bio-Sciences Corp). The protein concentration was determined using the BCA assay (Pierce), and purity was monitored in 15% polyacrylamide gel electrophoresis (SDS-PAGE). Endotoxin was removed after successive washing steps with 1% Triton X-114 (19). Endotoxin levels of the preparations, determined by Chromogenic Limulus Amebocyte Lysate assay (LAL) (Cambrex Biosciences), were equal to 0.1 EU of endotoxin per 1 μg of purified protein.

### Cloning, expression, and purification of LTs

Construction of the pML19, pML21, and pLTK63 plasmids encoding the LT1 (GenBank Accession Number GI 408994), LT2, and LTK63 toxins, respectively, was carried out as previously described (9) and using data available at GenBank for LT1 and showed by Imamura and coworkers (17) and Pizza and coworkers (12). We constructed LTK63, in which serine at position 63 of the A subunit was replaced by lysine, at the background of LT1. The LT2 natural variant has five polymorphic sites in the A subunit (S190L, G196D, K213E, S224T, and N238D) and one in the B subunit (T75A) compared to LT1 (9, 16). Toxin purification by affinity chromatography on immobilized d-galactose columns (Pierce) was described previously (9). Endotoxin levels of the preparations determined by LAL assay were equal or lower to 0.2 EU of endotoxin per 1 μg of purified protein.

### SDS-PAGE and immunoblot analyses

Proteins separated on 15% polyacrylamide gels were stained with Coomassie Blue R250 or transferred to nitrocellulose membranes. Immunoblots for the detection of the p24 protein were carried out using standard procedures with anti-HIV p24 rabbit serum (NIH AIDS catalog no. 4250) diluted in 1% bovine serum albumin (BSA) in PBS (block solution).

### Functional characterization of purified LT

Determination of the intracellular adenosine cAMP levels was performed using the Y-1 adrenal tumor cell line, as previously described (20). The GM1-ELISA was carried out using standard procedures (21). The binding of LT to mouse Y-1 cells was determined using 1 μg of the tested toxin at 4°C for 1 h in DMEM medium. Treated cells were washed with PBS containing 1% fetal bovine serum and incubated with mouse anti-LT serum. After 30 min, the cells were stained with a FITC-conjugated anti-mouse IgG antibody (Invitrogen) and analyzed by flow cytometry (FACS Calibur, Becton Dickinson, Mountain View, CA, USA). The data were analyzed using the FlowJo program (Tree Star).

### Transfection of cells and immunofluorescence analyses

The specificity of the antibodies raised in mice immunized with the recombinant HIV-1 p24 protein was determined with A3.01 cells transfected with the pHXB2 plasmid encoding the complete genome of HIV-1 (18). Transfection with the pHXB2 plasmid encoding the complete HIV-1 genome was carried out with polyethylenimine (PEI) (1 mg/mL) (Polysciences) for 30 min at 37°C in a 5% CO_2_ atmosphere. After 7–8 days, transfected cells were harvested, added to 96-well plates (Nunc), washed twice with PBS, and fixed with methanol at room temperature. The plates were then incubated with blocking solution (0.2% Tween-20, 3% skim milk in PBS) for 30 min at 37°C before being probed with primary antibodies for subsequent immunofluorescence labeling. The plates were exposed to primary antibodies (sera derived from mice immunized with the recombinant p24 protein plus Complete Freund’s Adjuvant or PBS) diluted to 1:500 for 1 h at 37°C. After two washings with PBS, the plates were incubated with FITC-labeled goat anti-mouse IgG (Invitrogen) plus 1 μg/mL DAPI (Invitrogen) for 1 h at 37°C. Cells were washed twice with PBS, dried at room temperature, and visualized with an immunofluorescence inverted microscope (Nikon) equipped with a digital camera.

### Migration of dendritic cells to draining lymph nodes and *in vivo* activation

BALB/c mice were inoculated with intradermal (i.d.) or subcutaneous (s.c.) injections of 1 μg of LT1, LT2, or LTK63, and the inguinal lymph nodes (ILNs) were removed 24 h after the immunization. Pooled ILNs cells were harvested after tissue homogenization with a glass homogenizer and extensive washing with 2% fetal bovine serum (FBS)/PBS. Cells were plated at a concentration of 5 × 10^6^ cells per well and stained with anti-CD11c and anti-I-A^d^ (MHC-II), plus anti-H-2K^d^ (MHC-I), anti-CCR7, anti-CD80, anti-CD86, or anti-CD40 conjugated antibodies (BD Biosciences). Labeled cells were suspended in PBS containing 2% FBS and analyzed (1.5 × 10^6^ events/sample) in a cell cytometer (FACSCanto, Becton, Dickinson and Company). The data were analyzed using FlowJo software (Tree Star) gated on the CD11c^+^/MHC-II^+^ cells. Results were measured in pooled ILNs cells harvested from mice subjected to the different treatments (*n* = 5 per group). All samples were tested in duplicates, and each tested condition was repeated independently twice.

### Mice and immunization protocols

Specific pathogen-free BALB/c mice (8–15 weeks old) were supplied by the Isogenic Mouse Breeding Facility of the Department of Parasitology, Institute of Biomedical Sciences, University of São Paulo (USP). All animal handling was carried out in accordance with the principles of the Brazilian code for the use of laboratory animals and was approved by the committee on the ethical use of laboratory animals at the Institute of Biomedical Sciences, USP. Mice were immunized with 20 or 100 μL aliquots administered by i.d. or s.c. injections, respectively, on the dorsal surface on days 0, 14, 28, and 42. For the i.d. injections, the mice were partially shaved on the dorsum 1 day before injection. Purified p24 protein was injected at a dose of 20 μg/animal admixed with 1 μg of LT1, LT2, or LTK63. Control groups received sterile PBS or non-adjuvanted p24 protein. Serum samples were collected 1 day before each immunization for ELISA assays. Animals were sacrificed 2 weeks after the last immunization for the determination of the CD4^+^ and CD8^+^ T cell responses and the *in vivo* CTL activity. The experiments were performed independently three or two times, respectively with *n* = 5 animals per immunization group as indicated on respective legend.

### Local skin inflammatory effects

BALB/c mice were i.d. inoculated with a single dose of 1 μg of LTs per animal/20 μL at previously shaved dorsal caudal area. Local skin reactions were inspected regularly for redness, edema, and swelling for 10 days after injection. The diameter of swelling was measured with a caliper. The experiments were performed independently twice with *n* = 5 animals per immunization group.

### Detection of anti-p24 serum antibody responses

p24-specific ELISA was carried out with 96-well Maxisorp plates (Nunc International) coated with purified p24 protein (5 μg/mL) diluted in PBS at pH 7.5 and incubated overnight at 4°C. Serum samples from immunized mice, anti-HIV p24 rabbit serum (NIH AIDS catalog no. 4250) or serum from an HIV-infected individual (NIH AIDS catalog no. 192) was serially diluted (twofold) in blocking solution and added to the wells for 1 h at 37°C. Reactions were performed with diluted horseradish peroxidase-conjugated rabbit anti-mouse immunoglobulin (total IgG, IgG1, or IgG2a) (Sigma and Southern Biotech), anti-human IgG antibody (Southern Biotech), or rabbit anti-IgG antibody.

### Detection of secreted cytokines

Secreted cytokines were measured using the ELISA BD OptEIA™ kit (BD Biosciences) with the supernatants of cultured spleen cells harvested from immunized mice. The cells were stimulated with a synthetic peptide (GenScript) corresponding to the I-A^d^ CD4^+^ T cell-specific (AMQMLKETINEEAAE) epitope of the HIV-1 p24 protein at a final concentration of 5 μg/mL for 48 h at 37°C in a 5% CO_2_ atmosphere. After stimulation, the supernatants were collected for determination of the IFN-γ and IL-5 concentrations. All samples were tested in duplicate, and each immunization experiment was repeated independently twice.

### ELISPOT assay

The IFNγ-ELISPOT was performed essentially as described earlier (22). Spleen cells collected from immunized animals were diluted in complete media, plated at a concentration of 5 × 10^5^ cells per well, and incubated for 24 h at 37°C in a 5% CO_2_ atmosphere in medium with and without the synthetic peptide corresponding to the major H-2K^d^ CD8^+^ T cell-specific (AMQMLKETI) p24 epitope at a final concentration of 2.5 μg/mL. All samples were tested in duplicate, and each immunization experiment was repeated independently three times.

### *In vivo* CTL assays

The *in vivo* CTL responses were determined as previously described (22). Briefly, splenocytes collected from naive BALB/c mice were stained with 0.5 μM (CFSE^low^) or 5 μM (CFSE^high^) carboxyfluorescein diacetate succinimidyl ester (CFSE) (Invitrogen Molecular Probes). The CFSE^high^ cells were pulsed for 40 min at 37°C with 1 μM of the synthetic p24 CD8^+^ T cell-specific peptide. Subsequently, aliquots containing equal amounts of CFSE^low^ and CFSE^high^ cells (2 × 10^7^ cells) were intravenously injected into mice previously immunized with the different vaccine formulations and naïve control mice. Twenty hours later, the numbers of CFSE^low^ and CFSE^high^ cells were determined in the spleens of the recipient mice using flow cytometry. The percentage of specific cell lysis was determined for each animal using the formula 100 − [(% CFSE^high^ in immunized mice/% CFSE^low^ in immunized mice)/(% CFSE^high^ in naïve mice/% CFSE^low^ in naïve mice)] × 100%. All samples were tested in duplicate, and each immunization experiment was repeated independently twice.

### Statistical analyses

One-way ANOVA followed by Tukey’s HSD test was used to compare the differences between the mean values of the immunization groups. Differences with *p* ≤ 0.05 were considered statistically significant. The statistical analyses were performed with the animals belonging to each immunization group (*n* = 4–5) with independent experiments.

## Results

### Generation of the recombinant p24

**Figure 1:**
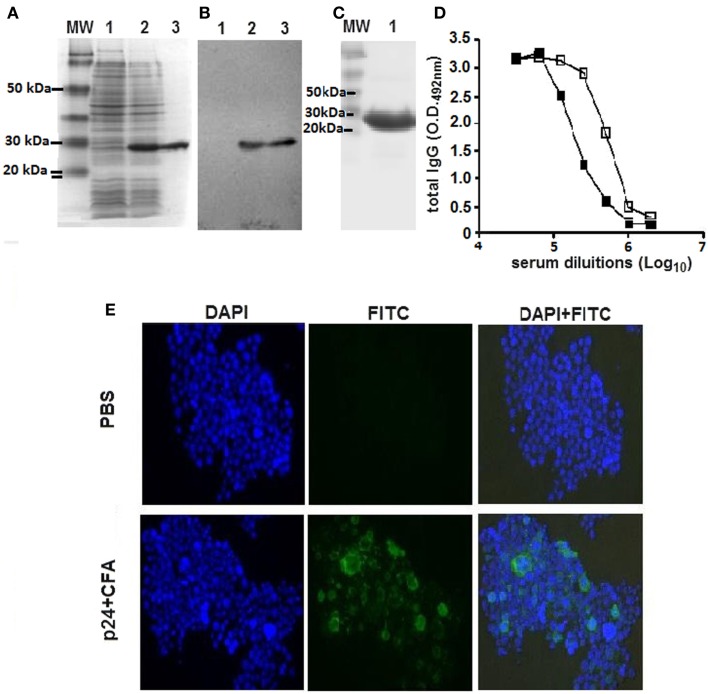
**Purification and immunological characterization of the recombinant HIV-1 p24 protein**. **(A)** SDS-PAGE analysis of whole-cell extracts of the recombinant bacterial strain and purified p24 protein. Samples: lane 1, whole-cell extract of the *E. coli* BL21(DE3) strain transformed with the plasmid encoding the recombinant p24 cultured without addition of the inducer; lane 2, whole-cell extract of the *E. coli* BL21(DE3) strain transformed with the plasmid encoding the recombinant p24 cultured after addition of the inducer; lane 3, purified p24 after affinity chromatography in a nickel-containing resin. MW, PageRuler™ pre-stained Protein Ladder (Fermentas). One to two lines were loaded with 10 μL of whole-cell extract of *E. coli* at D.O._600nm_ 4 before (line 1) or after (line 2) IPTG addition or 3 μg of purified p24 protein (line 3); **(B)** Immunoblot developed with p24-specific rabbit polyclonal serum. Protein samples were the same as described above. **(C)** SDS-PAGE analysis of purified p24 protein not completely separated on 15% polyacrylamide gels. **(D)** Antigenicity of the recombinant p24. The protein was employed as solid phase-bound antigen in ELISA plates developed with sera obtained from an HIV-1 infected subject (open squares) or from a rabbit immunized with recombinant p24 (closed squares). **(E)** Antibodies raised in mice immunized with the recombinant p24 recognize the native viral protein. Cells of the A3.01 lineage were transfected with the pHXB2 vector encoding the complete HIV-1 genome. After 7 days, the cells were fixed and incubated with serum from mice immunized with p24 combined with Freund’s adjuvant (lower panel series) or PBS (upper panel series), at a final dilution of 1:500. Cells were labeled with FITC-conjugated anti-mouse IgG antibody and DAPI. The experiments were performed twice showing the same results.

A soluble recombinant form of the HIV-1 p24 protein was generated in bacterial cells purified by nickel affinity chromatography (Figure 1A). The recombinant protein retained the antigenicity of the native viral protein, as demonstrated in Western blots and ELISAs developed with reference serum samples collected from a rabbit immunized with purified p24 protein and an HIV-infected subject (Figures 1B,D). The recombinant protein p24 were at least 95% pure (Figure 1C) and contained 0.1 EU of endotoxin per microgram of purified protein. Additionally, anti-p24 antibodies raised in mice immunized with the recombinant p24 recognized the native virus protein expressed by A3.01 cells transfected with a plasmid vector encoding all virus proteins (Figure 1E).

### Generation and biological characterization of LTs

**Figure 2:**
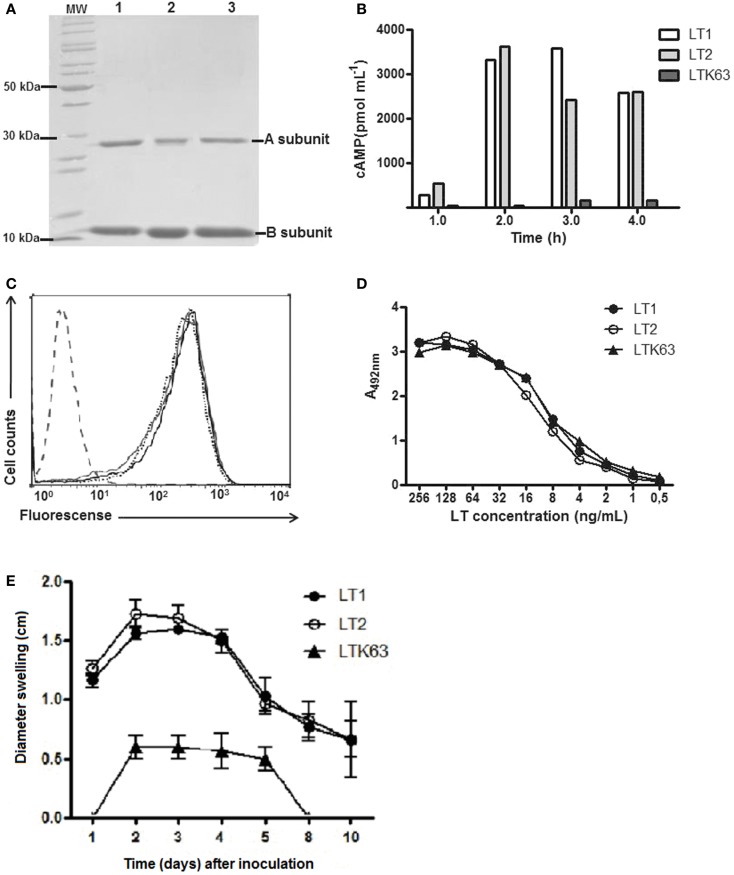
**Purification and functional characterization of the recombinant LT derivatives**. **(A)** SDS-PAGE of the purified recombinant LT (2 μg/lane). Samples: lane 1, LT1; lane 2, LT2; lane 3, LTK63; and MW, PageRuler™ Unstained Protein Ladder (Fermentas). **(B)** *In vitro* cAMP accumulation in Y-1 cells exposed to trypsin-activated LT1, LT2, or LTK63 for different time periods. **(C)** Binding of the purified LTs to Y-1 cells. Cells were incubated with LT1, LT2, or LTK63 for 1 h and labeled with mouse anti-LT and FITC-conjugated anti-mouse IgG antibodies before analysis of emitted fluorescence in a flow cytometer. Samples: LT1 (dotted line); LT2 (black line), and LTK63 (gray line). Untreated Y-1 cells are indicated by the dashed line on the left side of the figure. **(D)** Receptor binding activities of the purified LT derivatives in GM1-ELISA. Reactions were developed with mouse anti-LT serum and peroxidase-conjugated anti-mouse IgG antibody. **(E)** Local edema formation in BALB/c mice (*n* = 5) after i.d. injection of 1 μg of the test LT forms. The experiment was independently performed three times.

The three LT derivatives (LT1, LT2, LTK63) were expressed and purified from recombinant *E. coli* strains. The recombinant toxins displayed similar electrophoretic motility, binding to purified GM1 receptor and Y-1 cells (Figures 2A,C,D). In addition, LT1 and LT2 displayed similar enzymatic activity, as demonstrated by the increase in intracellular cAMP in treated Y-1 cells (Figure 2B). Mice inoculated by the i.d. route with LT1 and LT2 developed intense local inflammatory reactions measured by edema formation and induration at the inoculation site as early as 24 h post administration and persisted for more than 10 days. In contrast, mice inoculated with LTK63 induced a slight swelling reaction at the injection site detected at 48 h after inoculation and vanished 8 days later (Figure 2E). Similar reactions were observed in mice inoculated via the s.c. route (data not shown).

### Activation of migratory behavior and *in vivo* activation of DC in LT-inoculated mice

**Figure 3:**
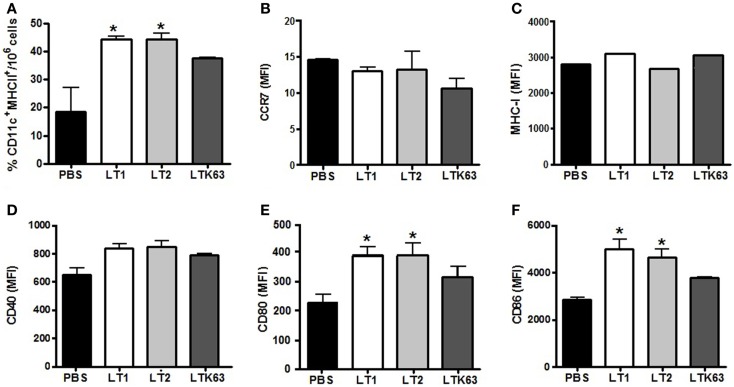
**Effects on DC migratory behavior and the expression of activation markers after the administration of the LT derivatives via the i.d. route in BALB/c mice**. Draining ILNs were collected 24 h after injection of 1 μg of the tested LT derivatives, after which CD11c^+^/MHC-II^+^ cells were analyzed in a flow cytometer. **(A)** Total number of CD11c^+^/MHC-II^+^ cells detected after injection of the tested LT; **(B–F)** Median fluorescence intensity of CD11c^+^/MHC-II^+^ cells labeled for **(B)** CCR7, **(C)** MHC-I, **(D)** CD40, **(E)** CD80, or **(F)** CD86 markers. The expression of surface markers was analyzed by acquiring 3 × 10^6^ events. Asterisks indicate statistically significant differences (*p < 0.05) with regard to the control mouse group (PBS). Results are expressed as means ± SD of two experiments performed independently with pooled of ILNs cells harvested from mice (*n* = 5) subjected to the different treatments.

We also tested the *in vivo* effects of the LTs on the behavior of CD11c^+^/MHC-II^+^ **dendritic cells** (DCs) after administration via the s.c. and i.d. routes. As shown in Figure 3, 24 h after a single i.d. administration of the tested LTs, the numbers of DCs at the ILNs were significantly higher among LT-treated mice than PBS-treated animals. Mice inoculated with the non-toxic LTK63 showed a lower number of CD11c^+^/MHC-II^+^ cells in the draining lymph nodes than mice inoculated with LT1 or LT2 (Figure 3A). Similar results were observed in mice immunized via the s.c. route (data not shown). However, no significant difference in the expression of CCR7, a marker associated with DC migratory behavior (23), was observed in mice treated with the LTs (Figure 3B). We also measured the *in vivo* activation of CD11c^+^/MHC-II^+^ cells in ILNs after inoculation of the different LTs. Although no significant increase in the expression of MHC-I and CD40 (Figures 3C,D) was detected in mice treated with the LT derivatives, CD11c^+^/MHC-II^+^ cells harvested from mice inoculated with LT1 or LT2 showed an enhanced expression of CD80 and CD86 compared to the control group (Figures 3E,F). Similar results were also observed in mice inoculated via the s.c. route (data not shown).

### Adjuvant effects of LT derivatives on B cells and CD4^+^ T lymphocytes in mice immunized via parenteral routes

**Figure 4:**
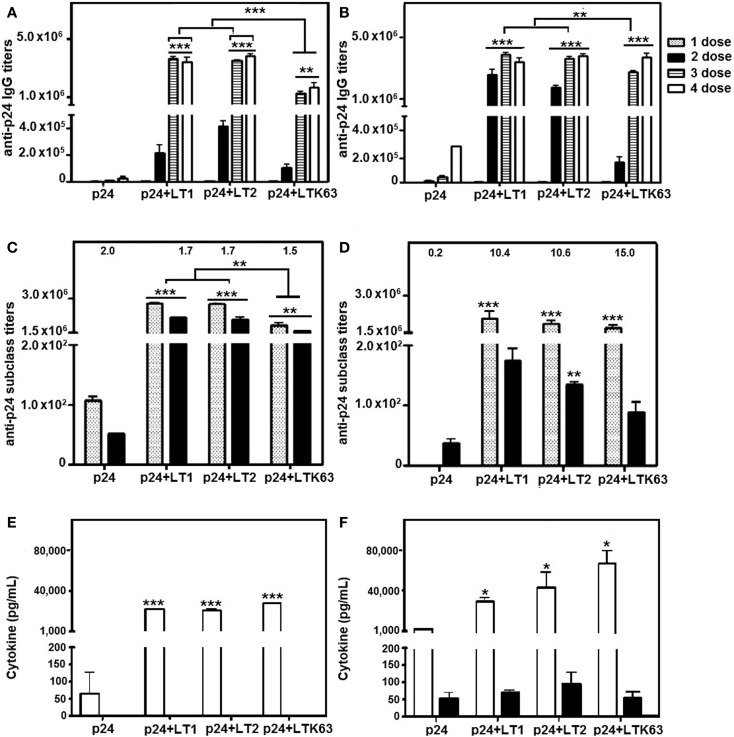
**Antigen-specific serum IgG and secreted cytokine responses in mice immunized with p24 adjuvanted with LT derivatives via i.d. or s.c. routes**. BALB/c mice were immunized with four doses of p24 admixed with LT1, LT2, or LTK63. Two weeks after each dose, mice were bled to obtain p24-specific serum. A control group was immunized with purified p24 without adjuvant. **(A,B)** Anti-24 IgG titers were measured in mice immunized via i.d. **(A)** or s.c. **(B)** routes. **(C,D)** IgG subclass responses elicited in mice immunized via i.d. **(C)** or s.c. **(D)** routes after the fourth dose. The numbers on the top of the columns represent the IgG1 (dotted bars)/IgG2a (black bars) ratio for each group. **(E,F)** IFN-γ (white bars) and IL-5 (black bars) secreted by spleen cells collected from mice immunized via i.d. **(E)** or s.c. **(F)** routes 2 weeks after the last immunization. Spleen cells were stimulated *in vitro* for 20 h with a synthetic peptide (AMQMLKETINEEAAE) encompassing the immunodominant p24-specific MHC-II-restricted CD4^+^ T cell-specific epitope. Results are expressed as means ± SD (*n* = 4–5 per immunization group) of three **(A–D)** or two **(E,F)** experiments performed independently. Statistically significant differences with regard to the p24-immunized mouse group are indicated with asterisks above the columns. In brackets, comparisons were carried out between different LT-treated groups. *p < 0.05; **p < 0.01; ***p < 0.001.

Titers of p24-specific serum IgG were increased in mice immunized via s.c. and i.d. routes with the recombinant p24 protein admixed with LT1, LT2, or LTK63. No statistically significant differences were observed in the anti-p24 antibody titers in mice treated with LT1 or LT2 following either i.d. or s.c. immunization (Figures 4A,B). However, lower anti-p24 IgG titers were detected in mice immunized with i.d. injections of LTK63 compared with mice immunized with LT1 or LT2 (Figure 4A). Analysis of the IgG subclass responses elicited in LT-vaccinated mice showed that, after four vaccine doses, mixed IgG1/IgG2a response was observed among animals regardless of the route of immunization. However, higher IgG1/IgG2a ratios were detected in mice immunized via s.c. route compared to mice immunized via the i.d. route (Figures 4C,D).

Immune response evaluations of cytokine profiles showed that splenic **CD4^+^ T cells** of mice immunized with i.d. injections of the LT derivatives secreted similar levels of IFN-γ and undetectable amounts of IL-5 (Figure 4E). Nonetheless, following s.c. vaccination, mice immunized with LTK63 showed higher levels of IFN-γ secretion by CD4^+^ T cells than mice treated with LT1 or LT2, whereas differences in the IL-5 levels were not observed between any of the immunization groups (Figure 4F). IL-4 was not detected in the culture supernatants of splenic cells from mice vaccinated via either the i.d. or the s.c. route (data not shown). Additionally, all LT forms increased IFN-γ levels compared with p24 administered alone, following either i.d. or s.c. immunization. These results, together with the IgG subclass findings, show that LT derivatives modulate the anti-p24 response toward a more biased Th2 profile when administered via the s.c. route compared to mice immunized via the i.d. route.

### CD8^+^ T cell adjuvant effects of LT derivatives in mice immunized via parenteral routes

**Figure 5:**
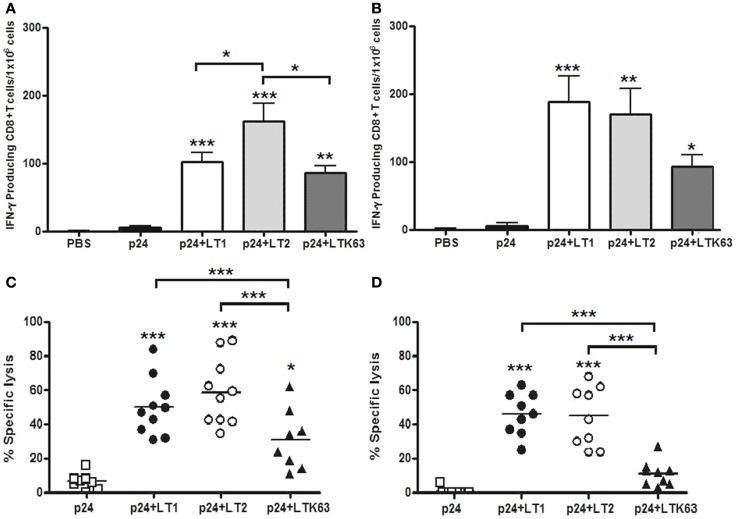
**CD8^+^ T cell adjuvant effects of LT derivatives administered via i.d. and s.c. routes**. **(A,B)** IFN-γ ELISPOT assay carried out with spleen cells of mice immunized with four doses of p24 adjuvanted with LT1, LT2, or LTK63 or p24 non-adjuvanted. Spleen cells were harvested from animals immunized via i.d. **(A)** or s.c. **(B)** routes 2 weeks after the last vaccine dose. Cells were stimulated with a synthetic peptide encompassing the p24 immunodominant MHC-I-restricted CD8^+^ T cell epitope for detection of IFN-γ-secreting cells. Results are expressed as means ± SD (*n* = 4–5 per immunization group). Experiments were performed independently three times. **(C,D)** *In vivo* CTL responses in mice immunized with LT via i.d. **(C)** or s.c. **(D)** routes. Two weeks after the final immunization, mice were injected with splenic syngeneic CFSE-labeled cells pulsed with the AMQMLKETI peptide. The specific *in vivo* CTL responses were determined 20 h later by the CFSE^high^/CFSE^low^ cell ratios, as described in the Section “Materials and Methods.” Results are expressed as means ± SD (*n* = 4–5) of two experiments performed independently. Statistically significant differences with regard to the p24-immunized mouse group are indicated with asterisks above the columns. In brackets, comparisons between different LT-treated groups. *p < 0.05; **p < 0.01; ***p < 0.001.

Adjuvant effects on CD8^+^ T cells were measured in spleen cells harvested 2 weeks after the last immunization dose. IFNγ-ELISPOT results showed that mice immunized with the LT derivatives induced strong activation of p24-specific CD8^+^ T cells, whereas no significant response was detected in mice immunized with non-adjuvanted p24. Mice immunized with LT2 by the i.d. route showed statistically higher CD8^+^ T cell responses compared to mice immunized with LT1 or LTK63 (Figure 5A). In contrast, mice immunized with LT2 via the s.c. route showed similar CD8^+^ T cell responses compared to mice immunized with LT1 (Figure 5B). The results also showed that the LT derivatives enhanced *in vivo* CTL responses after administration either i.d. or s.c. immunization. Nonetheless, mice immunized with LTK63 mounted significantly lower *in vivo* CTL responses than mice immunized with LT1 and LT2 (Figures 5C,D).

## Discussion

In the present study, we investigated the adjuvant effects of a natural LT form (LT2) after administration of a soluble antigen (a recombinant HIV p24 protein) administered via i.d. and s.c. routes to mice. The LT2 derivative has been detected in several ETEC strains isolated from both diarrheic and asymptomatic subjects representing the second most frequent LT polymorphic variant found among clinical-derived ETEC strains (9, 16, 17). LT2 proved to be endowed with strong adjuvant effects similar to those detected in mice immunized with the reference LT form (LT1) and superior to those induced by LTK63 with respect to antigen-specific serum antibody responses and activation of CD8^+^ T cell and CTL responses. LT1 and LT2 also promoted the more efficient *in vivo* activation of DC, as evaluated both by migration to local lymph nodes and expression of co-stimulatory molecules (CD80 and CD86). Altogether, these data indicate that LT2 preserves both the biological and immunological features of the reference LT1 toxin representing alternatives for vaccine adjuvants delivered via s.c. and i.d. routes.

The adjuvant effects of LT have been intensively investigated for more than three decades. Previous studies were carried out with native or non-toxic derivatives of a reference LT form (LT1) usually administered via mucosal routes (3, 6, 9, 20). The general conclusion from these studies is that LT derivatives act as powerful adjuvants both for serum and mucosal antigen-specific antibody responses and may lead to different T helper cell activation patterns that may change according to the nature of the antigen, attenuating mutations, and administration route (2, 8, 24–30). Our results based on the serum antibody responses induced in mice support previous evidences indicating that the humoral adjuvant effects of parenterally delivered LT are not dependent on the toxic activity of the A subunit (4, 8, 31). Moreover, IgG subclass response and cytokine secretion patterns of splenocytes led us to conclude that an enzymatically active A subunit does no significantly change the activation of antigen-specific CD4^+^ T cell responses. On the other hand, s.c. administration of LT derivatives significantly changed the induced immune response to a more biased Th2 response. This result led us to investigate the activation of DCs in mice following exposure to different LT derivatives.

The enzymatic activity of LT2 affected the activation of mouse DCs, as demonstrated by the migratory behavior to the draining lymph nodes and enhanced expression of activation markers (CD80 and CD86). DCs have the unique ability to internalize protein antigens, migrate into draining lymph nodes, process and present processed epitopes to naïve T lymphocytes. The adjuvant activity of LT derivatives, similarly to that of cholera toxin and other adjuvants, involves the recruitment and activation of DCs, as demonstrated both under *in vitro* and *in vivo* conditions (24, 27, 28, 30, 32). Our results confirmed that, at the same concentrations, LT1 and LT2 more efficiently activate DCs than LTK63, further supporting the role of the ADP-ribosylation adjuvant activity. The enzymatic activity of the A subunit was also linked to the local transient inflammatory reactions observed in mice inoculated with LT derivatives. Mice inoculated with LT1 or LT2 developed local edema that persisted for some days after administration. Such local reactions would probably contribute to the more efficient activation of DCs and should not represent a serious obstacle for future clinical testing, as demonstrated by the testing of LT using the transcutaneous route (10, 11). Administration of LT1 and LT2 via the s.c. or i.d. routes would the inherent higher adjuvanticity devoid of the risks involved with the mucosal administration of these toxins.

The present study demonstrated unequivocally that LT2 promoted high activation of CD8^+^ T lymphocytes as measured by the number of IFN-γ producing T cells. The number of IFN-γ producing CD8 T cells correlated with the activation of antigen-specific CTL response suggesting that LT2, similarly to LT1, promote the generation of multifunctional CD8^+^ effector T lymphocytes. Inversely, mice immunized with LTK63 elicited a significant lower activation of IFN-γ-producing CD8^+^ T cells and *in vivo* CTL responses. Such differential adjuvant activity may correlate with the lower migration of DCs for ILNs and lower expression of costimulatory molecules observed in mice inoculated with LTK63 with regard to LTs with preserved enzymatic activity. Additionally, these results indicated that, concerning activation of functional CD8^+^ T cell responses, preservation of enzymatic activity of LT has a relevant role in this adjuvant effect regardless of the administration route.

Extensive clinical and experimental evidence supports the notion that the activation of antigen-specific cytotoxic CD8^+^ T cells is required for the efficient control of HIV or SIV infections by promoting the clearance of infected cells (33–37). There is no doubt that an efficient anti-HIV vaccine will require the coordinate activation of B cells as well as CD4^+^ T and CD8^+^ T cells. Previous reports have demonstrated that both toxic and non-toxic LTs can induce antigen-specific CD8^+^ T cell responses following mucosal administration (20, 38–41). The present results indicate that LT2 shows strong adjuvant activity for a complete spectrum of immune responses, particularly for functionally active CD8^+^ T cells following administration via i.d. and s.c. routes. Our results add further evidences that parenteral delivered LT-adjuvanted vaccines may represent a valuable alternative for activation of CD8^+^ T cell responses, particularly for diseases caused by viruses such as HIV.

## Conflict of Interest Statement

The authors declare that the research was conducted in the absence of any commercial or financial relationships that could be construed as a potential conflict of interest.

## Glossary

Adjuvant: Vaccine adjuvants are broadly defined as all components that have functional ability to enhance *in vivo* immunogenicity of the antigens. Basically there two categories: delivery systems, that mainly function to co-localize vaccine components and to target vaccines to antigen-presenting cells (APCs), as DC; and immune potentiation. That directly activates immunological cells, particularly APCs, through specific receptors.
CD4^+^ T cells or T helper: The CD4^+^ T cells act of the activation, growth, and survival of the other cell types, such as DC, B, and T cells. Further more, these cells promote both changes on the antibody isotypes and long-term memory. These cells secrete cytokines and chemokines that modulate the immune response to bias Th1 or Th2 favoring humoral or cellular, respectively. CD8^+^ T response antigen requires CD4^+^ T cell help.
CD8^+^ T cell-dependent cytotoxic response: Activated CD8^+^ T cells mediate their effector functions by direct cytotoxicity and by secretion of cytokines such as IFN-γ. Rapid death of target cells is induced by perforin-mediated transfer of granzymes that initiate apoptosis. Slower killing is caused by Fas ligand signaling to cells that express Fas. These cells are pivotal for the clearance of a wide variety of pathogens and tumors.
Dendritic cells: Dendritic cell are bone marrow-derived cells of rare frequency but wide distribution in peripheral tissues. DC constantly sample their environment but not necessarily initiating an immune reaction until activation by a danger signal causes maturation, a state characterized by increased expression of surface MHC and co-stimulatory molecules, that results in increased ability to stimulate T cells.
Non-toxic derivatives of a reference LT: Site-directed mutagenesis permitted the generation of mutations at the active site and resulted in full or partially non-toxic LT forms which conserved adjuvanticity at the mucosal level. Among these mutants, LTK63 shows a complete knockout of the enzymatic active after substitution of serine to lysine at position 63 of the A subunit.
